# Editorial: Evolution, emerging functions and structure of actin‐binding proteins, Volume II

**DOI:** 10.3389/fcell.2023.1329219

**Published:** 2023-11-08

**Authors:** Lei-Miao Yin, Dmitri S. Kudryashov, Christos G. Zervas, Kai Murk

**Affiliations:** ^1^ YueYang Hospital, Shanghai University of Traditional Chinese Medicine, Shanghai, China; ^2^ Department of Chemistry and Biochemistry, The Ohio State University, Columbus, OH, United States; ^3^ Center of Basic Research, Biomedical Research Foundation, Academy of Athens, Athens, Greece; ^4^ Institute of Biochemistry, Charité Universitätsmedizin Berlin, Berlin, Germany

**Keywords:** actin-binding proteins, cytoskelcton, actin, transgelin-2, smooth muscle cell, muscle cell

Since the discovery of actin in the skeletal muscle in the 1940s, it has been found as a major cytoskeletal component mediating a wide range of cellular processes in all eukaryotic cells. In fulfilling these functions, actin is assisted by over 400 actin binding proteins (ABPs) (Gao and Nakamura, 2022), which can be generally divided into two classes: those that regulate the G-actin/F-actin cycle and those that organize actin filaments in higher order structures (Yin et al., 2021; Rajan et al., 2023). The number of proteins involved in the actin cytoskeleton organization is even larger, as many interact with ABPs rather than actin itself. Such proteins can be called actin-cytoskeleton associated proteins (or actin-associated proteins, AAPs). Furthermore, the actual complexity of the actin cytoskeleton cannot be fully appreciated without realizing that many ABPs and AAPs are present in several isoforms, while their activity is altered, often dramatically and unpredictably, by numerous post-translational modifications (PTMs) (Yin and Schnoor, 2022). Still, numerous mutations in actin, ABPs, and AAPs distort the actin cytoskeleton’s functionality, leading to genetic diseases that affect skeletal and heart muscle, immune, neural, bone, connective, and other tissues. Therefore, a not-so-uncommon opinion that the actin cytoskeleton is well understood is far from being accurate, as new functionalities of ABPs (e.g., processive pointed end actin polymerization; Kudryashova et al., 2022) are discovered regularly, while most are not sufficiently understood.

Similarly, the therapeutic potential of ABPs has been barely tapped. In recent years, many ABPs have been considered as promising therapeutic targets in various diseases, such as cancer and asthma (Yin et al., 2019; Dahlstroem et al., 2023). For example, Wiskott-Aldrich syndrome protein (WASP) and WASP-interacting protein are tumor suppressors in T cell lymphoma; therefore, corresponding inhibitors could have a potent therapeutic effect (Menotti et al., 2019). Transgelin-2 regulates pulmonary resistance in asthma, and its agonists could aid in treating asthma (Yin et al., 2018). Outside of the cell, the diagnostic and therapeutic potential of plasma gelsolin (an ABP involved in Ca^2+^-dependent actin remodeling in the cell and actin sequestering in the extracellular space) in detecting inflammation and ameliorating consequences of generalized thrombosis have been revealed (Piktel et al., 2018; Zhang et al., 2023).

The current special issue contains 10 manuscripts, five original research works and five reviews, that address recent advances in understanding of the various aspects of ABP organization and function. Thus, Iyer and others employed a comprehensive analysis of protein–protein interaction databases to reveal a network of 2482 AAPs (Iyer et al.). Three other manuscripts of the issue focused on PTMs of ABPs. Thus, Lin et al. focused on L-plastin, the immune cell-specific isoform of a calponin-homology (CH) actin-bundling protein. While -L-plastin is dispensable for phagocytosis, its replacement with a phosphorylation-impaired S5A mutant paradoxically impaired phagocytosis, likely by affecting the recruitment of vinculin to phagosomes (Lin et al.). Whereas phosphorylation of Ser406 activates L-plastin by alleviating the inhibitory association between actin-binding domains (Schwebach, et al., 2022), the mechanisms behind plastin activation by S5-phosphorylation remain poorly understood and should be clarified in future studies. Cornelius et al. reports that disruption of profilin2a interaction with AAPs and signaling lipids in neurons via its phosphorylation at Ser147 is essential for basal synaptic actin dynamics, dendritic spine remodeling, and long-term potentiation and depression processes (Cornelius et al.). F-actin destabilization via a less conventional, reversible PTM, MICAL-mediated oxidation of actin’s Met44 and Met47 (Mox-actin), is reviewed by Rajan et al. This work systematically describes the MICAL family along with enzymes involved in the reversal of the modification, early reports on the direct effects of this PTM on the actin filament stability, and recent studies on the contribution of other ABPs to this regulation.

In addition to the L-plastin study mentioned above, several other manuscripts focus on actin cross-linking and bundling proteins, reflecting a continuous rise of interest in high-order actin assemblies. Thus, the work by Mirouse reviews the evolution and functions of dystrophin (another CH-domain organizer) beyond its recognized role in the organization of adhesion complexes in the muscle by comparing phylogenetic and functional data of dystrophin-associated protein complex between different vertebrate and invertebrate models (Mirouse). The role of calcium-dependent actin cross-linking proteins in orchestrating mechanical forces in various cell processes and particularly in cell migration and wound closure, is reviewed in Lehne and Bogdan. The evolution, regulation, and function of the isoforms of vertebrate calponin and transgelin in controlling cell motility and contraction are comprehensively summarized by Hsieh and Jin. Xu et al. reviewed the recent studies on the modalities of ezrin regulation and its involvement in the biological processes of female reproductive physiology (Xu et al.). An original research study by Yang et al. characterized the largest isoform of human ectoplasmic specialization protein (espin 1) ectopically expressed in *E. coli*. The authors mapped the actin-binding site of espin-1 using a co-sedimentation assay and detected the protein on actin bundles using negative staining transmission electron microscopy with Ni-NTA-nanogold particles (Yang et al.).

On unexpected properties of ABPs whose function was thought to be well understood, Ono et al. identified a low molecular weight isoform of *C. elegans* tropomyosin (LEV-11U) produced along with six other products of the same gen as the result of alternative splicing. Despite the fact that LEV-11U interacted poorly with actin and, accordingly, was diffusely localized in the cytoplasm of the striated muscle, its expression was found to be important, implying a possibility for a novel biological function of this tropomyosin (Ono et al.).

In summary, this special issue collects timely research reports and several detailed reviews that contribute to a vast task of detailed characterization and better understanding of ABP functions in health and disease. New isoforms of known ABPs are discovered as the result of these efforts, sophisticated effects of PTM are described, and previously unknown structural features of several ABPs are identified. We sincerely hope that this special issue will aid in filling the knowledge gaps in building a comprehensive picture depicting the role of ABPs and AAPs in the organization of the actin cytoskeleton at large - in different organisms, tissues, cells, and subcellular locations.
